# An alternative to current psychiatric classifications: a psychological landscape hypothesis based on an integrative, dynamical and multidimensional approach

**DOI:** 10.1186/1747-5341-9-12

**Published:** 2014-07-17

**Authors:** Thomas Lefèvre, Aude Lepresle, Patrick Chariot

**Affiliations:** 1 Department of Forensic Medicine, Hôpital Jean-Verdier (AP-HP), avenue du 14 juillet, F-93140 Bondy, France; 2 Inserm, UMR 1101, LaTIM – Laboratory of Medical Information Processing, CHRU Morvan, Brest, France; 3 Institut Mines-Telecom, Telecom Bretagne, Image and Information Processing Department, Brest, France; 4 Department of Psychiatry, CHRU Amiens-Nord, Amiens, France; 5 Institut de recherche interdisciplinaire sur les enjeux sociaux (IRIS), UMR 8156–997, UFR SMBH, Sorbonne Paris Cité, université Paris 13, Paris, France

**Keywords:** System dynamics, Classification, Psychiatry, DSM

## Abstract

**Background:**

Mental disorders as defined by current classifications are not fully supported by scientific evidence. It is unclear whether main disorders should be broken down into separate categories or disposed along a continuous spectrum. In the near future, new classes of mental disorders could be defined through associations of so-called abnormalities observed at the genetic, molecular and neuronal circuitry levels.

**Methods:**

We propose an alternative hypothesis to these classifications based on an integrative, dynamical and multidimensional approach.

**Results:**

We suggest that observed data collected in the general population can be used to build a psychological landscape. Innovative techniques issued from information processing and system dynamics can prove helpful in this task. Information preserving techniques can reduce the high dimensional data collected and provide an intrinsic map for psychological characteristics or behaviors. Dynamical patterns called attractors, which are linked to each other through continuous pathways, can be identified. Specific attractors can define mental disorders. Their causal structure can be investigated with causal networks.

**Conclusions:**

Powerful and reliable tools are available so that an alternative to current psychiatric classifications can be built based on a genuine biopsychosocial model. The proposed model is ready to be tested on real data.

## Introduction

There is no agreement so far on how best to define and diagnose mental illnesses. Since its third edition published in 1980, the Diagnostic and Statistical Manual of Mental Disorders (DSM) featured definitions of mental disorders that achieved reliability equivalent to that of most medical diagnosis [1]. The revised DSM-III-R edition abandoned the idea of a hierarchy between disorders and the DSM-IV showed the lack of clear separations between current disorders, enhanced by evidence of high levels of comorbidity [2]. The recent DSM-5 aims to keep pace with the science of psychiatry and to improve the field from clinical and public health perspectives [3]. Yet, DSM-5 will place disorders in discrete categories that are largely based on decades-old theories and subjective symptoms. Neuroscientists and geneticists have been unable to find any evidence to support the breakdown of complex mental disorders into separate categories [4] or to find associations between genes polymorphisms or neuronal functioning abnormalities and clinical diagnosis [5]. The Research Domain Criteria (RDoC) project considers mental disorders from a translational point of view. It incorporates an explicitly dimensional approach to psychopathology in terms of dysfunction of particular systems, as studied from an integrative, multi-systems point of view [6]. Some authors recently suggested that psychiatry should consider a dimensional, rather than categorical approach: mental disorders can be represented along a continuous linear spectrum, from mental retardation to mood disorders [4,7]. The biopsychosocial model built some 40 years ago [8] could be seminal for elaborating a relevant alternative to the DSM, which relies on a biomedical model.

### Hypothesis

It is possible to search for a global map of psychological characteristics, without any a priori about the connections between nosological entities. The map could be shaped by the relationships between psychological characteristics or behaviors. Classical statistical techniques would provide a flat map, with linear correlations between characteristics [9]. A flat linear space cannot describe such complex objects as mental states. We propose that the intrinsic complexity of the relationships linking various characteristics should be taken into account, without assuming that they are either linear or proportional. In epigenetics, the description of studied phenomena can preserve the complex intrinsic relationships between observations. Waddington assumed that a map called ‘the epigenetic landscape’ could describe in one single geometric description the distinct evolution of cells starting from their totipotent state [10]. The specific cell states, corresponding to differentiated and specialized cells, are steady states of the landscape, as well as basins of attraction. We hypothesize that the natural evolution of a person could be described according to their successive positions in a psychological landscape [Table 1]. This landscape would be shaped by personal and environmental factors and various life events taken altogether.

**Table 1:** Stepwise method to test the dynamical and multidimensional hypothesis of a psychological landscape

**Main steps**	**Step details**
1. Construction of a research protocol	Includes: 1) questionnaires searching for personal characteristics and basic symptoms; 2) other data collection at the personal level (imaging, genetics, blood samples); 3) data collection about the macro levels (e.g., data about the kind of neighborhood, rural or urban places of living, macro-economic data).
2. Sampling of the population	Representative sampling of the general population and building of a comprehensive dataset made of the different kinds of data mentioned in step 1.
3. Dataset dimensionality reduction	Reducing the dimensionality of the dataset with information preserving techniques so that a minimal space of description is built.
4. Intermediate dimensionality analyses	Intermediate dimensionality analyses of the space of description: identification of the main factors associated to the dimensions of the minimal space.
5. Construction of a minimum general questionnaire for primary care	Construction of a minimum general questionnaire to be used in primary care settings based on the previous dimensionality analysis. Answers given to this questionnaire allow locating approximately a person on the landscape.
6. Partitioning of the minimal space of description	Partitioning of the minimal space of description with clustering techniques. The regions obtained are termed nosological areas.
7. Searching for pathways between nosological areas	Search for natural continuous pathways linking these nosological areas and analysis of the critical parameters that lead to brutal changes in pathways. Causal parameters associated with these pathways can be analysed by the means of causal networks.
8. Searching for attractors in nosological areas	For each identified nosological area, search for attractors in the sense of system dynamics, and determine the shape and characteristics of these attractors. Each attractor is a steady psychological pattern, may it be termed as pathological or normal. An attractor associated with an impaired functioning or an overwhelming pain could be seen as a pathological pattern.
9. Secondary and local dimensionality analyses	For each identified nosological area, local analysis and determination of the local dimensionality of the area, so that minimal and specific questionnaires can be built. The questionnaires are intended to situate individuals on the landscape more accurately; they can be used in second intention, not in primary care settings.

## Methods

The building of such a landscape requires the use of specific and dedicated technical steps: 1) estimation of data complexity; 2) reduction of data dimensionality so that they can be tractable; 3) the identification of patterns in data; 4) the reconstruction of the fine structure of these patterns, termed attractors; and 5) the discovery of causal structures underlying attractors and of causal paths between patterns.

### Estimating data complexity: “true” dimensionality estimation

There is presently no consensual definition for complexity, and therefore no unique technique to estimate data complexity. Complexity is usually opposed to a reductionist and linear approach [11-14]. Linearity applies when a complex problem can actually be broken down into several smaller problems, which can be more easily and independently solved. The solution of the initial problem is then the superposition, i.e. the addition, of smaller problems solutions. Complexity rather lies in quality than in quantity. The examined relationships may not need as many parameters as the total number of measures. The measures may be partially redundant, overlapping and correlated to each other. To reduce the apparent complexity of initial data, we can search for the minimal number of parameters needed to properly describe the data without losing the core complexity. The complexity which needs to be preserved is that of the relationships between measures, and thus between persons: a person which is close to another one in reality must be close to the same person once initial data are reduced. It is a topological complexity. Several techniques are available to estimate the “true” dimensionality of data, e.g., maximum likelihood estimator or counting box estimator [15,16].

The search for the true dimensionality of data can be explained with a simple analogy. The surface of the earth is grossly spherical, so we represent the earth in a three-dimensional space. Nonetheless, we use maps, which are two-dimensional spaces, because we only need two dimensions to locate a person on the surface of the earth. The “true” dimension of the surface of the earth is then two and not three.

### Reduction techniques preserving the topology of data

It is known that such maps are not a perfect representation of the earth, because they imply geometrical distortions (either angular or distance distortions). A correct map can be retrieved that preserves relative distances between each pair of points.

**Figure 1:**
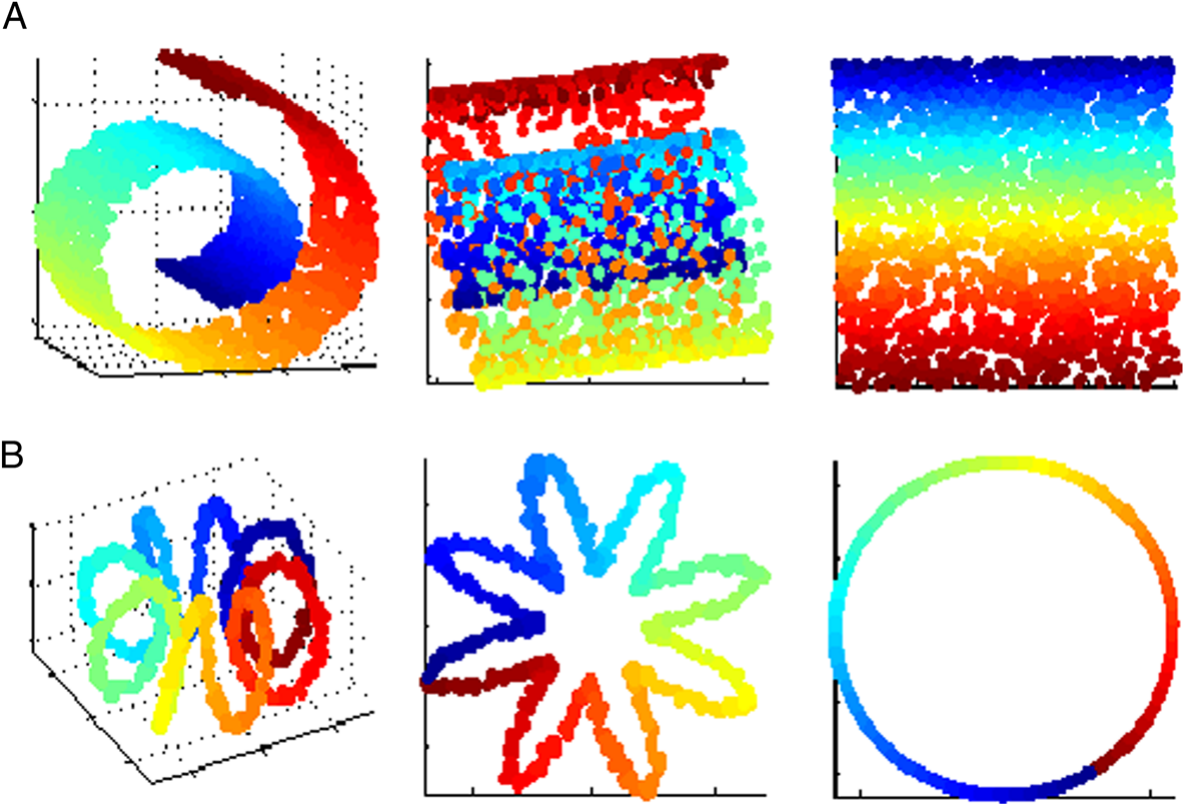
**Reduction of two complex datasets A (a roll) and B (a helicoidal tore) with two methods, classical linear method (PCA) versus nonlinear method (ISOMAP).** PCA relies on projections: original data (left) are represented in a three-dimensional space. PCA projected data into a two-dimensional space along their principal axes and the neighboring relationships are not preserved (center). ISOMAP properly unfolds the data while preserving true neighbors (right). ‘True’ neighbors are dots of similar colors: the dimensionality reduction technique does not respect neighboring relationships when a red dot lies next to a blue dot.

When some complexity is preserved while reducing data, the exact form of the relationships does not need to be known but neighbors in the initial data must remain neighbors in the reduced data. Some nonlinear dimensionality reduction techniques can reduce complex data while preserving its topological structure. Linear approaches assume proportionality between associated factors: a statistical correlation between a factor A and a factor B is a linear correlation, which merely implies that B = I.A, where I is a constant. A way to define complex data is to assume that relationships between factors may be more complex than linear relationships. For instance, U-shaped relationships are known and can be exhibited, e.g., the relationship between healthcare costs and age. Costs are maximal for extreme ages and minimum at mid-life. Even relationships as simple as U-shaped relationships cannot be automatically detected in data [9,17]. Classical linear reduction techniques such as PCA fail to preserve topological structures while NLDR techniques succeed (Figure 1).

### Pattern recognition techniques, robust clustering

There exists a wide range of techniques termed pattern recognition or clustering techniques all aiming at two distinct goals. They can either be used (1) to automatically classify events or observations after a proper training when a gold standard is available (e.g., the automated recognition of hand written addresses on postcards [18]), or (2) to retrieve homogenous forms in data, without any a priori knowledge and in the absence of a gold standard (e.g., the identification of distinct profiles of users of the healthcare system). Clinical researchers show a growing interest in the first kind of use, for instance to improve and automate diagnostic tests [19]. Psychiatrists also made recent attempts to link categories of diagnosis and neuro-imaging patterns by using these techniques [7]. This approach can be criticized since a gold standard is mandatory to train pattern recognition techniques (i.e. a test, whatever its nature, that can diagnose without any error a person with a specific nosological category). Such a gold standard does not exist. We prefer to use the pattern recognition technique so that no gold standard is needed and no strong assumption about the shape and structure of possible nosological categories is made. We only search for homogenous regions, gathering people on the basis of similar characteristics. The use of pattern recognition techniques implies the search for consistent homogenous groups, invariant under a set of perturbations. We suggest determining these groups with an approach based on their robustness [20]. A group can be termed robust, if most members are still grouped together after we randomly resample the whole population. Thus, groups are still composed of the same persons no matter the number of times or the proportion of them we shuffle. These groups are invariant forms of the data.

### Techniques for attractor reconstruction

A specific system may be characterised by the nature and the shape of its attractor rather than by a mean. The issue consists in identifying the shape of the attractors. According to Takens’ theorem, attractors can be retrieved in datasets [21] and reconstructed from experimental data [22]. In the biomedical area, the homeostasis theory due to Cannon and Bernard has been later provided with a theoretical framework as an implementation of the theory of systems and Wiener’s cybernetics theory [23,24]. In this framework, a so-called normal state is regulated by the system using a negative feedback and the integration of various signals.

The complex systems theory provides tools which derived from an extension of the cybernetics theory. It includes the long-term past of the system to process the present signals, whereas cybernetics operates in an absolute and never-ending present. As recently emphasized, the complex systems theory can contribute to identify common processes across different levels of human functioning. The regulation of internal states under unpredictably external circumstances, as observed in paranoia, is a good candidate for such a process [25].

### Reconstruction techniques for causal networks (Bayesian networks)

**Figure 2:**
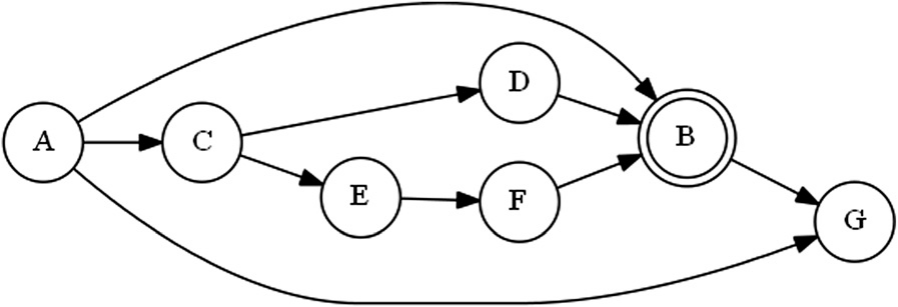
**A graphical representation of a Bayesian network.** Observations or factors are the nodes of the graph (e.g., A, B), and the arrows represent the link and the direction of the information linking two observations (A- > B: the knowledge of A implies probabilistically the knowledge of B. If A is tobacco smoking, B could be lung cancer). Data allow the structure of the graph and the probability associated to each arrow to be estimated.

The search for causal networks in data provides an alternative to the classical linear conception of causality: A causes or implies B. In linear approaches of causality, statisticians use multivariate analysis or multiple adjustments on covariates, seen as potential confounders for the causal association A/B. Most of these techniques rely on linear assumptions that restrain their validity [26,27]. The causal networks, such as Bayesian networks, evaluate the global structure of the data in terms of information propagation. In other terms, how does the knowledge of A imply the knowledge of B, C, D and so on? These networks do not rely on linear assumptions and tend to be more respectful of the complexity of the underlying relationships between observations. They have a graphical representation: a graph depicts the whole causal network, the observations (A, B…) being the nodes of the graph and the arrow being the probabilistic causal paths between observations (Figure 2) [28,29].

### Blueprint for a psychological landscape

#### Data reduction without complexity loss: from observations to the building of a raw map

**Figure 3:**
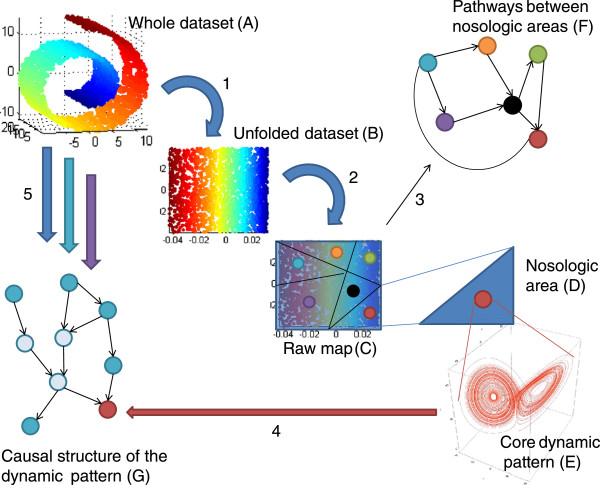
**Building the psychological landscape, from raw data to the determination and the causal analysis of its dynamical patterns. A**. The whole dataset is constituted of various data layers collected on persons, which include behavioral, biological, genetic, and imaging data. It may exhibit complex relationships that cannot be preserved or explored appropriately with classic statistical tools. **B**. Reduced dataset produced by nonlinear reduction techniques (NLDR), which allow minimal complexity loss (step 1). **C**. A raw map is produced by pattern recognition techniques and robust clustering (step 2). Homogeneous groups of persons may be identified, according to similar behaviors and psychological traits. Such groups are termed nosological areas. **D**. Zooming in on a specific nosological area. **E**. Applying algorithms for attractor reconstruction should allow the dynamic pattern of this area to be identified. **F**. The causal pathways leading to an attractor can be analysed with Bayesian networks or similar methods (steps 4 and 5). The links of the dynamic pattern to other items from the whole dataset, e.g., age and sex, can be also analysed. **G**. Once the main nosological areas are identified, pathways between these areas can be searched for. Bayesian networks can be used as well.

Building a map requires the collection of objective, subjective and contextual pieces of information. Some of these data are out of the scope of the main usual rating scales that are used in psychiatry, such as environmental, genetic and biological data. The underlying paradigm takes recognized basic features into account without assuming any hierarchy between neurobiological, psychological, and social dimensions in mental health. The initial description space will include as many dimensions as the number of distinct observations. Collected data become more heterogeneous when getting bigger. Big data are characterized by a lot of information and some of them, such as genetic and imaging data, are heavy, i.e. they gather more data than a classical library would do [30]. For example, imaging databanks are made of thousands of digital images, which are made of millions of colored pixels. Each pixel gives information to process. Collected data involve different aspects of a person and their environment. If the age, sex and height are the three dimensions used to identify a person, we can locate different persons relatively to each other according to these three parameters in a three-dimensional space. If other parameters are taken into account, e.g., symptoms, behaviors, biological or social data, we need a much higher dimensional space to locate persons relatively to each other. In such a space, the classical tools used to measure the distance or similarity between two persons are inoperative. All individuals tend to melt into a single and unique average person, who cannot be properly distinguished from another one. This phenomenon, which is known as the “curse of dimensionality” [31], hinders high dimensional datasets to be directly analysed with standard techniques. It is therefore necessary to reduce the dimensionality of the initial dataset to its lowest needed, while preserving the complexity of relationships between observations. The kind of complexity we are interested in here could be termed as a topological complexity: two persons close to each other in reality must remain close in the constructed map. The reduced dataset should preserve these neighboring relationships. Statistical techniques for dimensionality reduction are available, such as the principal components analysis (PCA) and PCA-like techniques [32]. These techniques should not be used because they may rule out any complex relationships between observations. The different kinds of collected data are different ways to observe and qualify a person. These data are linked to each other and present at least partially redundant or correlated information. A lower dimensional space can be studied, in which redundancy is dramatically reduced, e.g., when an initial three-dimensional space is reduced to a two-dimensional space. The reduced dimensionality is termed “true” or “intrinsic” dimensionality. The intrinsic dimensionality of a dataset can be approached by different techniques, e.g. those used in dynamical systems [15]. If the intrinsic dimensionality of the initial description space is known, more relevant techniques than PCA, e.g., nonlinear dimensionality reduction (NLDR) techniques, can be used to reduce dimensionality and recover the geometry of the map (Figure 3, step 1) without any loss of information [31,33]. Such techniques preserve proximity between similar persons or observations, regardless of the nature of their relationships.

#### Data analysis: global and local analysis of the psychological map

The search for specific regions starts once the map is built. These regions constitute parts of the map that naturally gather people sharing some characteristics, e.g., depressed persons sharing traits of sadness or anhedonia. Clustering techniques could be used to partition the map into specific homogeneous regions [18]. These techniques are widely used in several areas of research and industry [34], and currently contribute to identify the functions of “junk DNA” in the ENCODE project [35]. Hence a global map will be available with regions primarily defined from their local homogeneity and their robustness to minor changes (Figure 3, step 2). These regions can be termed “nosologic areas”. Each nosologic area should be thereafter more precisely investigated in order to encompass its dynamical behavior.

#### Analysis of the intrinsic variability of personal trajectories around attractors

The study of the dynamical features of the nosologic areas could benefit from the continuous advances of the theory of dynamical and complex systems. Evidence from the last two decades showed that the main physiological functions should be analysed with the same tools as in physics [36]. For instance, features of dynamical systems such as the cardiac rhythm should not be described with means and standard deviations, but according to their intrinsic variability. Thus a physiological state can be described by a steady pattern around which the measured parameter of a person will orbit. The various trajectories around this pattern in a specific region present an infinite intrinsic variability which cannot be reduced to a mean and a unique standard deviation. Such a pattern is named an *attractor* and trajectories are termed *orbits*.The attractor is located in a well-defined region of the map that virtually contains all possible combinations of characteristics leading to orbiting around this specific state. These infinite combinations are all confined to a finite region of what forms the psychological landscape (Figure 3, element D). Another remarkable characteristic of an attractor is that its shape is determined by a few critical parameters, if not only one. A change in value of these parameters can lead to the destruction or metamorphosis of the attractor. Attractors are therefore both quantitative and qualitative models that require careful characterisation.

#### Situating a person in the psychological landscape

**Figure 4:**
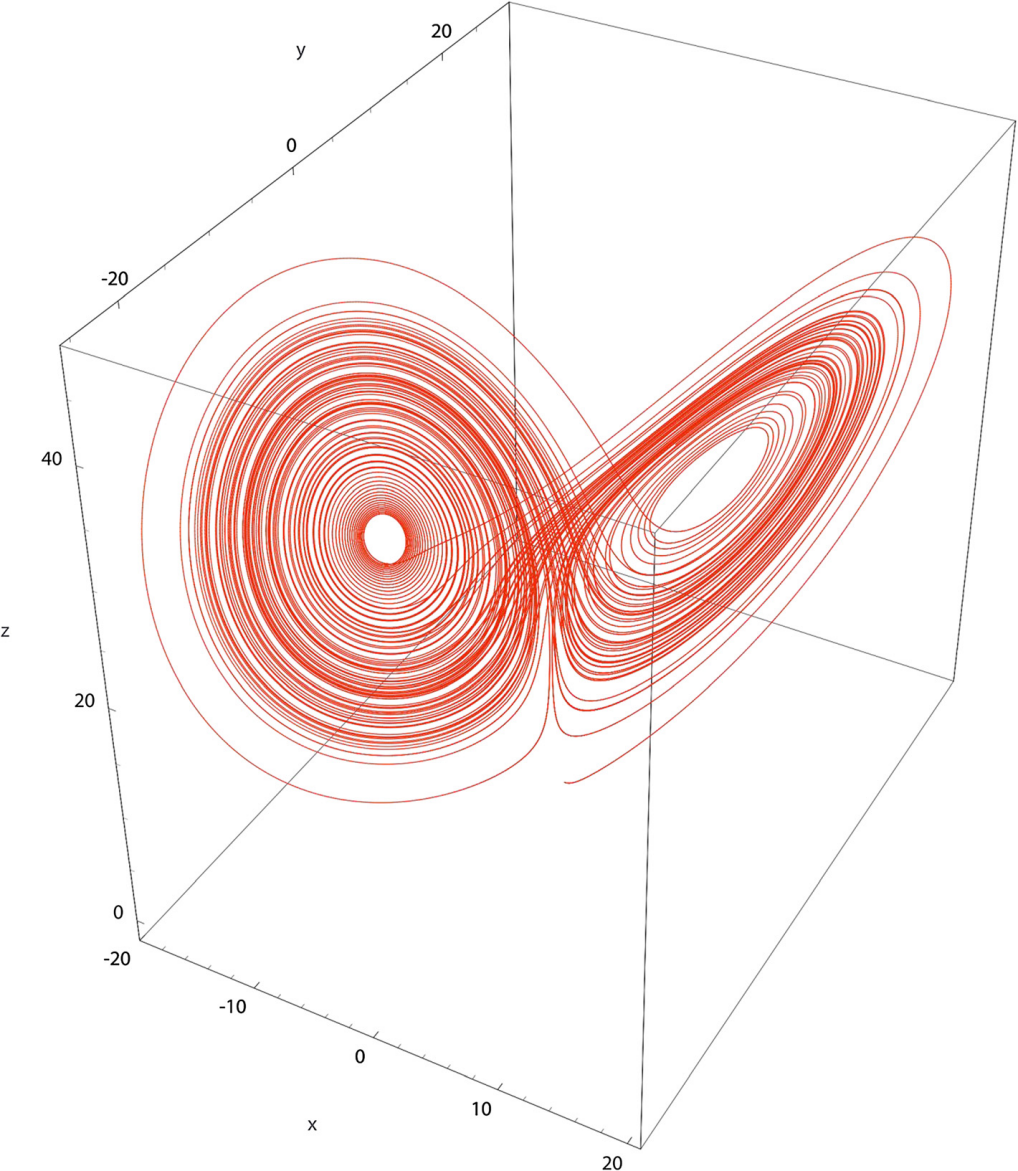
**A classical example of dynamic attractor: the Lorenz attractor.** It depicts the globally dynamical and stable behavior of some systems. It is made of individual trajectories that are captive and alternately orbiting around two distinct attracting poles.

The Lorenz attractor [37] could be used to describe the behavior of a person presenting alternately sadness and euphoria (Figure 4). In this case, the personal orbits would occupy that specific region of the psychological landscape including episodes of manic or depressive tones. Hence the trajectories would periodically orbit around two separate poles, which would be near or far from the core of the attractor (see Additional file 1). The distance from the core could be an illustration of the intensity of the manic and depressive traits of the episode. This distance could also signify the strength with which the person remains in this specific region, reflecting a more severe episode. Without any intervention from outside, or any change of the key parameters shaping the personal orbit, e.g., a treatment, this person could not exit from this region and would keep on orbiting around the same attractor.

While it is possible to fully characterize the structure of an attractor, i.e. to determine which factors shape it, the very dynamical nature of the attractor implies unpredictability at some particular level. The cited example is a possible representation of an alternating state between sadness and euphoria, whose transition cannot be satisfyingly predicted. Hence some individuals share the same global pattern but differ in terms of particular trajectories. The concept of landscape is relevant since it is possible to move inside, according to: 1) the inertia of the considered person and the obstacles on their way (i.e. it may be easier to recover from sadness to wrath, than from sadness to joy: in the first case, the pathway would be going downward, in the second case it would be going upward), 2) the events that may locally modify the landscape around the person, who could move from their position under the action of several factors, such as a treatment, a substance abuse, or a psychic trauma.

#### Causal networks: which pathways between nosological areas, which underlying structures for attractors?

According to the hypothesis that continuous pathways can be identified between nosological areas, we have to determine the structure of these pathways and which factors model them. Causal networks (Figure 2) are used to identify and to represent graphically the causal associations between factors. These causal pathways are probabilistic and rely on complex data relationships. No linear assumption about the factors is needed. Pathways between all possible combinations of nosological areas can be searched for (Figure 3, step 3).We can similarly determine the causal structure underlying an attractor (Figure 3, steps 4 and 5). In the case of the Lorenz attractor, the search for its causal structure would reveal the various components shaping and leading to this attractor, e.g., a specific brain activity, levels of inflammatory markers, or personal life events and environment. These factors are linked through probabilistic pathways, arranged in sequences. For example, the Lorenz attractor could represent a dynamical pattern in a nosological area gathering people with sadness and euphoria. Specific pathways could lead to this pattern: predisposing genes may be expressed and interact with traumatic life events, leading to changes in levels of inflammatory markers and impacting brain functionalities. The change of brain activity may also interact with personal history and habits, or coping strategies which can be exceeded.

## Conclusion

An atheoretical approach to improving the validity of psychiatric classification systems may be grounded on appropriate techniques. Reliable techniques currently exist which can support the rigorous integration of huge amount of data while preserving the real-world complexity, leading to the building of a psychological landscape. Existing databases provide only a limited amount of the data needed for the psychological landscape construction. Yet, the RDoC project, which integrates the fundamental genetic, neurobiological, behavioral, environmental and experimental components that comprise mental disorders, could provide a starting point for our hypothesis to be tested. The second British National Survey of Psychiatric Morbidity could serve as another useful basis [25], data should be linked to external surveys, e.g., national surveys, and participants re-contacted for additional details. A broad cooperation between health and social actors is required to set up the uniform collection of psychological, somatic, social and economical data from the general population.

## Abbreviations

DSM: Diagnostic and statistical manual of mental disorders; ENCODE: Encyclopedia of DNA elements; ISOMAP: ISOmetric feature MAPping; NLDR: Nonlinear dimensionality reduction; PCA: Principal components analysis; RDoC: Research domain criteria.

## Competing interests

The authors declare that they have no competing interests.

## Authors’ contributions

TL drafted the first versions of this paper, and provided the technical backgrounds and expertise. AL and PC revised each version. All authors read and approved the final version of the manuscript.

## Supplementary Material

Additional file 1 **The dynamical behavior of the Lorenz attractor.** As shown in the movie, the Lorenz attractor is made of potentially infinite trajectories around two distinct poles. Trajectories cannot be perfectly predicted, and different people may orbit around the same attractor, without sharing any trajectory in common. Note that this alternating phenomenon is not strictly periodic. Click here for file
